# Isolation and Characterization of a Novel *Actinomycete* Isolated from Marine Sediments and Its Antibacterial Activity against Fish Pathogens

**DOI:** 10.3390/antibiotics11111546

**Published:** 2022-11-03

**Authors:** Haimanti Mondal, John Thomas

**Affiliations:** Centre for Nanobiotechnology, Vellore Institute of Technology (VIT), Vellore 632014, India

**Keywords:** marine *Actinomycetes*, *Beijerinickia fluminensis*, antibacterial activity, FTIR, GC-MS

## Abstract

Marine habitats are especially complex, with a varied diversity of living organisms. Marine organisms, while living in such intense conditions, have developed great physiological and metabolic potential to survive. This has led them to produce several potent metabolites, which their terrestrial counterparts are unable to produce. Over the past few years, marine *Actinomycetes* have been considered one of the most abundant sources of diverse and novel metabolites. In this work, an attempt was made to isolate *Actinomycetes* from marine sediments in terms of their ability to produce several novel bioactive compounds. A total of 16 different *Actinomycete* colonies were obtained from marine sediment samples. Among the 16 *Actinomycete* isolates, 2 isolates demonstrated in vitro antibacterial activity against *Aeromonas hydrophila* and *Vibrio parahemolyticus.* However, among them, only one isolate was found to have potent antibacterial activity, and hence, was taken for further analysis. This isolate was designated as *Beijerinickia fluminensis* VIT01. The bioactive components obtained were extracted and later subjected to Fourier transform infrared spectroscopy (FTIR) and gas chromatography–mass spectroscopy (GC-MS) analyses for identification. Several novel bioactive compounds were reported from the data obtained and were found to have potent antibacterial activity. Hence, they could be used as an alternative to antibiotics for treating several fish pathogens in the aquaculture industry.

## 1. Introduction

*Actinomycetes,* belonging to the order Actinomycetales, are members of a heterogeneous group of Gram-positive bacteria, consisting of up to >55% GC content in their DNA [1]. They are anaerobic, with filamentous fungal morphology and branched growth patterns, resulting in either extensive mycelium or colony. Later, the mycelium may fall apart to form coccoid or rod-shaped forms. Most genera of *Actinomycetes* form a spore called sporangia or spore cases, found on the colony surface, aerial hyphae or maybe within the free environment. They exhibit diverse genetic, biological and functional activities and are a good source of many new secondary metabolites [2,3,4]. The sea has a vast treasure of resources. Due to this fact, marine *Actinomycetes* play a significant role by being a major part of it [5]. Apart from this reason, they differ from their terrestrial counterparts considerably because of distinct environmental changes prevailing in marine habitats [6,7].

The first marine *Actinomycete* was discovered in the year 1984 [4,8]. Since that period, many novel species of marine *Actinomycetes* have been found in aquatic environments worldwide [9,10,11,12,13]. In addition, a majority of the strains of *Actinomycetes* from the marine environment have been isolated from sediments [14]. The population size of *Actinomycetes* in the case of oceanic sediments has been reported to vary with various physicochemical parameters, such as pH, temperature, salinity, total organic carbon, pressure, etc. [4].

In general, *Actinomycetes,* and in particular, marine *Actinomycetes* have a vast range of applications in various fields. Be it in drug synthesis to cure certain diseases or biologically active products, synthesis of antibacterial, antiviral, antifungal as well as anticancer products, synthesis of enzymes, or their major role in biofouling, they have contributed a lot in various areas of the aquaculture industry [5]. Sharma et al. [15] reported the applications of *Actinomycetes* in bioremediation, cancer treatment and production of some valuable antibiotics, such as amphotericin, neomycin, vancomycin, chloramphenicol, novobiocin, gentamycin, nystatin, erythromycin, tetracycline, etc. They are also used as biocontrol tools, antifungal compounds, bio corrosion and as a source of agroactive compounds. The bioactive compounds extracted from these marine *Actinomycetes* possess different chemical structures and conformations, which hold the key to novel drug synthesis that, in future, may be potent enough to combat various resistant pathogens [16].

*Actinomycetes* are widely distributed in the marine environment, in marine species such as *Streptomyces*, *Nocardia* and *Micromonospora*. Their occurrence on the contact slides and dead marine algae suspended in the sea has been reported [17].

A study reported by Ref [18] revealed that some new marine *Actinomycetes* have produced a variety of bioactive metabolites. The antibacterial activity of fermentation products from *Actinomycetes* isolated from the marine environment also showed that several bioactive compounds had activities against vaccinia virus replication multidrug-resistant Gram-positive bacteria and cancer cells.

*Actinomycetes* contribute 70% of antibiotic sources and numerous non-antibiotic bioactive metabolites, including enzymes, enzyme inhibitors, anti-oxidation reagents, immunological regulators, etc. [19]. In other words, currently, almost two-thirds of the antibiotics developed are derived from *Actinomycetes*. Marine *Actinomycetes* have been reportedly studied for their efficacy as a potent novel antibiotic producer [20,21,22,23]. Although the use of several antibiotics has been banned in the aquaculture industry [24], they are still being used and are causing resistance. In this context, the aim of the current work was to isolate a novel bioactive compound from marine *Actinomycetes,* which is effective against pathogens in aquaculture.

## 2. Results

### 2.1. Isolation of Actinomycetes

A total of 16 different *Actinomycete* colonies were obtained from marine sediment samples. To isolate the *Actinomycetes*, specific agar medium Actinomycetes Isolation Agar (AIA) was used, since it was found to be good for the growth of actinomycetes. Some morphologically different strains of *Actinomycetes* were obtained from the sediment samples collected off the coast of Digha, West Bengal, India.

### 2.2. Characterization of Isolates

Among the 16 *Actinomycete* isolates, 2 isolates demonstrated in vitro antibacterial activity against *Aeromonas hydrophila* and *Vibrio parahemolyticus.* However, among them, only one isolate was found to have potent antibacterial activity, and hence, was taken for further characterization. This isolate was designated as *Beijerinckia fluminensis* VIT01.

#### 2.2.1. Screening of Potential Actinomycete Isolates

The antibacterial activity of the isolate was confirmed by primary screening using the cross-streak method. This was followed by secondary screening using the agar well diffusion method. The crude extract of *Actinomycete* isolates showed a zone of inhibition against the two bacterial pathogens tested. Among the two pathogens, *Vibrio parahemolyticus* showed better results when compared to *Aeromonas hydrophila.* In the case of primary screening using the cross-streak method, the zones of inhibition observed in the isolate against pathogens *A. hydrophila* and *V. parahemolyticus* were 11 mm and 13 mm, respectively. In addition, the zone of inhibition observed in the secondary screening of the isolate was also found to be better in *V. parahemolyticus* compared to *A. hydrophila*.

#### 2.2.2. Screening of the Potential Isolate in Comparison to the Antibiotic

Gentamicin was found to be sensitive to both pathogens *A. hydrophila* and *V. parahemolyticus,* showing a zone of inhibition of 11 mm and 12 mm in the case of both *V. parahemolyticus* and *A. hydrophila*, respectively, using the disc diffusion method. On the other hand, the *Actinomycete* isolate gave a better zone of inhibition compared to the antibiotic.

**Figure 1:**
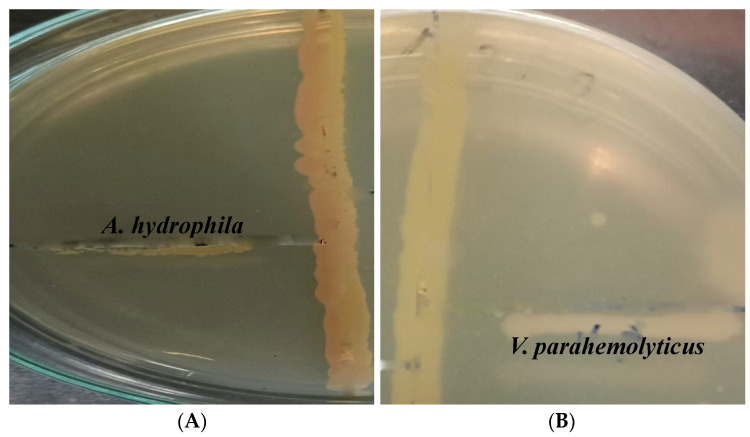
Primary screening of the antimicrobial activity of isolate 1 (*B. fluminensis* VIT01) against selected pathogens (**A**) *A. hydrophila* (**B**) *V. parahemolyticus*.

The zone of inhibition revealed in vitro antibacterial activity of *Beijernickia fluminensis* against both pathogens *Aeromonas hydrophila* and *Vibrio parahemolyticus,* as shown in Figure 1A. Figure 1B reveals the zone of inhibition of Gentamicin against *A. hydrophila* and *V. parahemolyticus*.

Table 1 summarizes the antibacterial activity of some *Actinomycete* isolates against *Aeromonas hydrophila* and *Vibrio parahemolyticus* compared to Gentamicin. The data table shows that compared to Gentamicin, *Beijernickia fluminensis* gave a better zone of inhibition against both pathogens *A. hydrophila* and *V. parahemolyticus.*

#### 2.2.3. Morphological Characterization

*Actinomycetes* were isolated, and their morphological appearance was observed. The staining results showed that the bacterium was Gram-positive, cocci-shaped, non-motile, capsulated and spore-forming.

##### FESEM Analysis

**Figure 2:**
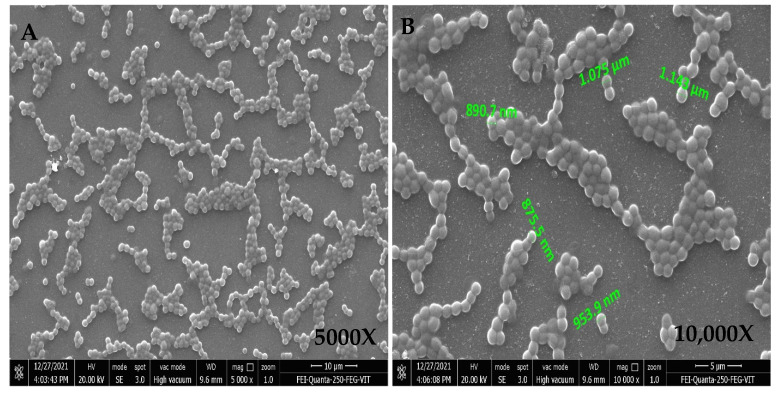
Scanning electron microscope images under 5000X (**A**) and 10,000X (**B**). Cocci in clusters.

The FESEM micrograph revealed the shape and arrangement of the isolate under different magnifications, as shown in Figure 2.

##### Biochemical Characterization

The biochemical tests performed showed a positive result for Gram-positive bacteria. The results indicate that the isolate was capable of starch, gelatin and casein hydrolysis. The results of biochemical tests are presented in Table 2.

##### Physiological Characterization

The tested *Actinomycete* isolate was able to grow in 1%, 3.5%, 5% and 7% NaCl concentration but showed resistance at 10% and 20% NaCl. The ideal temperature for the growth of *Actinomycetes* was found to be between 28 and 30 °C, although they could even grow in temperatures up to 40 °C.

##### Molecular Characterization

The 16S rRNA sequencing revealed that the strain belongs to *Beijerinckia fluminensis* under *Actinomycetes.* This was designated as *Beijerinckia fluminensis* VIT01.

### 2.3. FTIR Analysis of Crude Extracts from Actinomycetes

**Figure 3:**
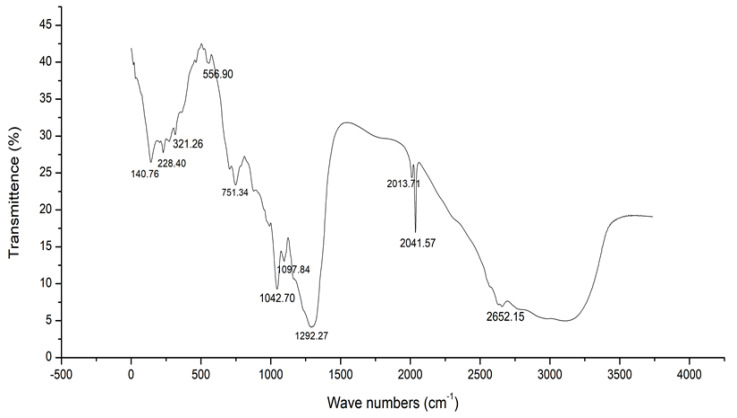
FTIR analysis of ethyl acetate extract of *Beijerinickia fluminensis*.

Figure 3 summarizes the FTIR spectra of the sample ranging from 400 to 4000 cm^−1^. The characteristic bands observed from Sample 1 are 2652.15, 2041.57, 1292.27, 1042.70, 751.34 and 556.90. The data shown in Table 2 reveal the vibrational type of the functional groups, their band strength and the compounds present in the respective peaks of the ethyl acetate extract of *B. fluminensis.* The peak at 2652.15 corresponds to the stretching vibrational type of weak thiol groups. The peak at 2041.57 was assigned to the stretching vibration of strong isothiocyanate groups. Similarly, the peaks at 1292.27 and 1042.70 correspond to the stretching vibration type of strong fluoro compound and medium amine groups, respectively. In addition, the peak at 751.34 corresponds to the bending vibration type of strong 1,2-disubstituted groups, and the peak at 556.90 corresponds to the stretching type of vibration of strong halo compound groups.

The peak values corresponding to their respective groups in the sample are shown in Table 3.

### 2.4. GC-MS Analysis of Crude Extracts from Actinomycetes

The GC-MS analysis of the identified active compounds isolated from the ethanolic extract of *Actinomycetes* displayed their peak percentage expressed with their retention indices in the chromatogram.

**Figure 4:**
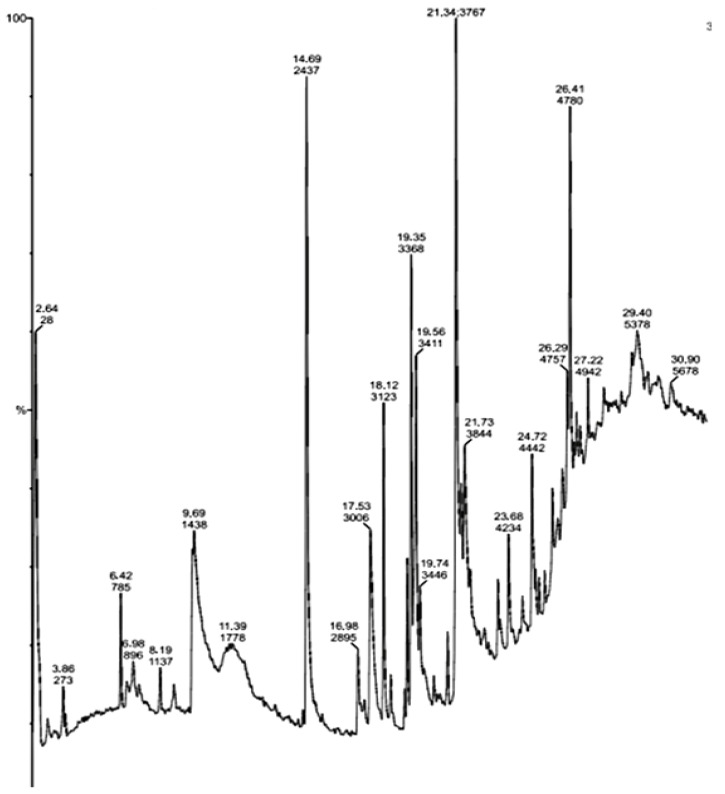
GC-MS chromatogram of ethyl acetate extract of *Beijerinickia fluminensis*.

The bioactive compounds identified from the ethanolic extract of *Beijerinckia fluminensis* VIT01 showed that they contain many active components. They are responsible for several properties, including antimicrobial and antibacterial, as per Dr. Duke’s Phytochemical and Ethnobotanical Database. Figure 4 represents the chromatogram of the bioactive compounds extracted from the ethanolic extract of the *Actinomycete* strain sample. The compounds are 1,6;3,4-Dianhydro-2-Deoxy-Beta-D-Lyxo-Hexopyrano; N,N-Dimethylheptanamide, Butanamide,3,N-Dihydroxy; (S)-Isopropyl Lactate; 3-O-Acetyl-exo-1,2-O-Ethylidene-Alpha-D-Erythrof; Glycine,N-Octyl-,Ethyl Ester; D-Glucitol,1-Deoxy-1-(Octylamino); Glycyl-L-Proline; 7-Tetradecene, (E); 2,3-Anhydro-D-Galactosan, etc. The data from Table 4 show the major peak, the retention time of the compounds and the activities present in the ethyl acetate extract of *B. fluminensis.* The results revealed that compounds such as N, N-Dimethylheptanamide, Glycine,N-Octyl-,Ethyl Ester, Glycyl-L-Proline, Actinomycin C2, (S)-3,4-Dimethylpentanol and 7-Tetradecene, (E), have antibacterial properties. Therefore, these compounds can be used as potent antibacterial agents.

Table 4 summarizes the main compounds, retention time, major peak area and major activities present in the sample. These bioactive compounds were later used for in vivo and in vitro studies.

## 3. Discussion

*Actinomycetes* have proven to be one of the best sources of secondary metabolites, and they have potent bioactive compounds [26]. Currently, the incidence of infectious diseases in the aquaculture industry is increasing, leading to significant loss in the aquaculture industry. Hence, there is a dire need to develop novel bioactive compounds that can be effective against various pathogens. While screening for *Actinomycetes*, various fast growing microbial colonies, which have the ability to inhibit the colonization of *Actinomycetes* on their particular isolation medium, were detected. In order to isolate *Actinomycetes* and prevent the growth of unwanted as well as ubiquitous bacteria, pre-treatment of the sediment samples was preferred. Gebreyohannes et al. [27] reported that potent antibiotic-producing actinomycetes isolated from the marine sediment were subjected to physical pre-treatment methods to stimulate the growth of *Actinomycetes*. In the present study, similar aseptic methods were followed to prevent the growth of unwanted bacteria.

In recent times, there has been a growing interest in the isolation of various novel and potent bioactive *Actinomycetes* from the marine environment, since they contain bioactive compounds. Many culture-independent studies [28] have reported that the marine environment contains a vast and rich diversity of rare *Actinomycetes.* They have been useful in designing successful isolation schemes to isolate a wide variety of marine *Actinomycetes.* Oggerin et al. [29] isolated *Beijerinickia fluminensis* strains UQM 1685^T^ and CIP 106281^T^ from soil. Similarly, a recent study was conducted by Shwaaiman et al. [30] who isolated a novel bacterium *Beijerinickia fluminensis* BFC-33 from soil. In the present study, we isolated *Beijerinickia fluminensis* from marine sediments. **Based on the literature survey, this might be the first report on the isolation of *Beijerinickia fluminensis* from marine sediments.**

Trakunjae et al. [31] reported that they isolated a rare *Actinomycete, Rhodococcus* so. BSRT1-1, which was Gram-positive, non-motile, with short rods that later converted to cocci. Oggerin et al. [29] isolated *Beijerinickia fluminensis* from a soil sample. In the present work, the bacterium *Beijerinickia fluminensis* was isolated from marine sediments. The present work revealed that the isolated bacterium is light orange colored. It is Gram positive, with cocci in clusters, and non-motile. This corroborated the study of Oggerin et al. [29] who reported that a white and cream colored isolate was non-motile. Several studies reported on the biochemical and physiological characteristics of *Actinomycetes*. A study by Ref [32] reported the biochemical and physiological characteristics of *Actinomycetes.* They reported the presence of starch and gelatin. Casein hydrolysis was also reported. They also reported negative results for indole, Voges–Proskauer, urease and lipase, and positive results for methyl-red and citrate. In the present study, *B. fluminensis* gave positive results for starch, casein and gelatin hydrolysis, citrate and urease, while negative results were obtained for methyl-red, Voges–Proskauer and indole tests. Rajkumar et al. [33] isolated actinomycetes from different environments. They reported that the isolated *Actinomycetes* showed negative Indole, MR and VP. They also reported that the isolates from *Actinomycetes* CAHSH-2 and *Actinomycetes* CAHSH-3 were positive for citrate, urease, starch, casein and gelatin. This is in agreement with our report. In the case of physiological characterization, Sivanandhini et al. [32] revealed that the *Actinomycetes* were able to survive in high salt concentrations. Similarly, our present work showed that *B. fluminensis* was able to tolerate up to 7% salt concentration. Another study reported by Ref [27] on the *Actinomycetes* isolated from water and sediments showed starch and urea hydrolysis. It was also able to tolerate NaCl concentration up to 5%. Our present study showed that both starch and urea were able to hydrolyze. It was also able to survive in 7% salt concentration. Similarly, Undabarrena et al. [34] reported the physiological characteristics of several species of Actinobacteria. They reported that they were able to survive at 1%, 3.5%, 5%, 7% and 10% NaCl concentration. In the present work, we observed that they were able to tolerate up to 7% salt concentration. Rajkumar et al. [33] also reported that the *Actinomycetes* were able to survive in 9% NaCl. In the present study, equally similar biochemical and physiological characteristics, such as NaCl tolerance (salinity), temperature tolerance, as well as biochemical characteristics of *Actinomycetes,* were reported.

Primary and secondary screening was performed to evaluate the antimicrobial potential of the microorganism against the pathogens. Singh et al. [35] screened thirty-six *Actinomycete* isolates against both Gram-positive and Gram-negative bacterial pathogens, such as *Staphylococcus aureus* MTCC 96, *Bacillus subtilis* MTCC 441 and *Escherichia coli* MTCC 64, and a fungal pathogen *Candida albicans* MTCC 183. Among them, fifteen showed strong and moderate antimicrobial activity. Later, they were subjected to secondary screening, where out of fifteen active isolates, thirteen exhibited strong antimicrobial activity. Another study by Ref. [36] reported that different strains of *Streptomyces* sp. showed antibacterial activity against *Vibrio* spp. In the present study, good antibacterial activity was seen in the primary screening of the isolate, which exhibited a zone of inhibition of 11 mm and 13 against pathogens *A. hydrophila* and *V. parahemolyticus*, respectively, followed by secondary screening.

Norouzi et al., 2018 [37] reported the antibacterial activity potential of several *Actinomycete* isolates against many bacterial pathogens in comparison to antibiotics such as Gentamicin using the Kirby–Bauer disc diffusion method. Antibiotic sensitivity test and disc diffusion were carried out to compare the activity of the crude extract of the *Actinomycete* isolate with the antibiotic Gentamycin. The disc diffusion revealed that, among the three isolates, two potent marine *Actinomycetes* isolates MN2 and MN39 were able to produce biomolecules with antibacterial activity against multidrug-resistant (MDR) bacteria. In our present study, we observed that our isolate gave a better result compared to Gentamicin. Gentamicin exhibited zones of inhibition of 11 mm and 12 mm in the case of both pathogens, *V. parahemolyticus* and *A. hydrophila.* The zones of inhibition observed in the isolate against pathogens *A. hydrophila* and *V. parahemolyticus* were 11 mm and 13 mm, respectively.

Currently, the number of many novel secondary metabolites reported from marine *Actinomycetes* has surpassed the number of their terrestrial counterparts [38]. Dholakiya et al. [39] reported the results of a study where the bacteria *Streptomyces variabilis* exhibited a wide spectrum of antibacterial activities against several Gram-negative and Gram-positive bacteria. Merochlorin E and F isolated from a marine bacterium *Streptomyces* sp. displayed antibacterial activities against several bacteria, such as *Staphylococcus. aureus, Bacillus. subtilis* and *Kocuria. rhizophila* [40,41]. Voon et al. [42] reported the FTIR spectrum of biocellulose from *Beijerinickia fluminensis* WAUPM53 in different media. Another study by Ref [43] reported the FTIR spectra of melanin produced by *Beijerinickia fluminensis.* In the current study, several potent antibacterial compounds were found in the FTIR and GC-MS analyses of *Actinomycetes.*

## 4. Materials and Methods

### 4.1. Sample Collection

The samples for screening were collected in the month of May 2021, off the coast of Digha, West Bengal, India. The marine sediment samples were collected from Digha (21°37′35.82″ N latitude and 87°30′26.75″ E longitude) and transferred to autoclavable bags and then stored in cell frost at 4 °C [27,34].

### 4.2. Media Used

#### Media

The Actinomycetes Isolation Agar (AIA) medium (Hi Media) was used for the isolation of *Actinomycetes*. Nalidixic acid (50 µg/mL) and Mycostatin (100 µg/mL) were added to the medium. The International Streptomyces Project (ISP-4) medium (TM media, Delhi, India) was used for purification of the isolates [44]. The Mueller–Hinton Agar (MHA) medium (HiMedia, Mumbai, India) was used for the determination of antimicrobial activity of the *Actinomycete* isolates.

### 4.3. Pre-Treatment of Sediment Samples

To enable the isolation of *Actinomycetes*, samples were subjected to pre-treatment. Sediment samples were kept overnight at 70 °C in a hot air oven for drying [45]. They were crushed and ground aseptically using mortar and pestle under aseptic condition.

### 4.4. Isolation of Samples

From the pre-treated sediment sample, 1 g was weighed and dispersed into a 100 mL conical flask containing distilled water with 0.9% saline and shaken continuously at room temperature using an orbital shaker for 10 min. This was considered as the stock culture for the sediment samples. A volume of 1 mL was taken from the stock solution and transferred to 9 mL sterile test tube containing water and mixed vigorously to reach a dilution factor of 10^−1^. Afterward, serial dilutions from 10^−2^ to 10^−6^ were carried out.

After serial dilution, a 0.1 mL aliquot sample of each dilution from 10^−3^ to 10^−6^ was plated in the AIA medium (HiMedia) containing 0.9% NaCl using the spread plate technique. The medium was amended with 50 µg/mL of Nalidixic acid (HiMedia) and Mycostatin (100 µg/mL) (HiMedia) to prevent the growth of other microorganisms. The Petri plates were incubated at 30 °C for 7–14 days until visible colonies were observed [39,46].

### 4.5. Screening of Samples

The pure colonies were counted using a colony counter. Their morphological characteristics and pigmentation were recorded.

After isolation, the isolates were screened and sub-cultured by individual streaking into AIA medium prepared with 0.9% NaCl. They were then transferred to new Petri plates containing AIA medium to obtain pure colonies. In addition, they were also sub-cultured and maintained in AIA slants at 4 °C [47]. The isolated bacteria were later stored at −20 °C in 20% glycerol [34,48].

### 4.6. Characterization of Isolates

Strains were identified based on morphological, cultural, biochemical characteristics and molecular analysis using 16S rRNA sequencing.

#### 4.6.1. Morphological Characterization

The selected isolates were streaked into an ISP4 medium to study their morphological characters by the macroscopic method. The colonies, morphology of the substrate and spores, aerial hyphae, branching and pigment production on the plates were observed in the preliminary stage. Gram staining, motility test and capsule staining were carried out to check the structure and motility of the isolated colonies.

##### Gram Staining

Gram staining was performed to identify the isolates, i.e., whether they are Gram positive or Gram negative. Later, the organism was first observed under 10× and 40× magnification and then under 100× with oil immersion [49].

##### Motility Test

A motility test was carried out by inoculating the isolate using the Sulphide Indole Motility (SIM) medium with an inoculation loop up to half or one-third inch above the bottom in a straight line. It was then incubated at 30 °C for 7 days. In addition, it was also observed under 10× and 40× magnification through the microscope to check its motility.

##### Capsule Staining

For capsule staining, Nigrosin was used as the primary stain and crystal violet as a counterstain. It was observed under 100× oil immersion microscope.

##### Field Emission Scanning Electron Microscope (FESEM) Analysis

The isolates selected were examined microscopically for spore chain morphology under 3000×, 5000×, 8000×, 10,000× and 13,000×. FESEM analysis was performed by two different methods to check the arrangement of the spores, the sporulating structures and the external surface morphology of the isolates. In the first method, the isolate was inoculated in a broth medium and incubated in a rotary shaker at 30 °C for 7 days until there was growth of bacteria. The broth culture was centrifuged at 7000 rpm for 10 min, and the supernatant was discarded. An amount of 1 mL autoclaved 1× Phosphate Buffer Saline (PBS) was added to the cell-free supernatant (CFS) and mixed well. Later, 10–20 µL of this culture was transferred to a clean grease-free slide. An amount of 20 µL of 0.25% Glutaraldehyde solution was dispensed into the slide containing the culture and oven dried overnight at 40 °C. In the second method, a clean, grease-free slide was taken, and a thin smear of the isolate was made in the center of it, air dried completely and kept overnight in a hot air oven at +40 °C.

#### 4.6.2. Biochemical Characterization

##### Biochemical Tests

Biochemical tests, such as ONPG, Ornithine, Lysine, Phenylalanine, Urease, H_2_S Production, Nitrate Reduction, Methyl Red–Voges–Proskauer’s Tests (MR-VP), Indole Production, Esculin Hydrolysis, Malonate, Catalase, Oxidase, Citrate Utilization, Crystal violet, Casein Utilization and Growth in MacConkey Agar, were carried out using a readily prepared KB003 Hi 25 Kit (HiMedia). Both Strip-I and Strip-II reaction wells of the kit were inoculated with *Actinomycete* culture using an inoculation loop incubated at 30 °C for 7–14 days.

In addition to the above-conducted tests, readily prepared KB009 Hi Carbo^TM^ Kit B009C HiMedia kit was used to check the capability of the isolated *Actinomycetes* to use several carbon compounds as an energy source. The kit consisted of 11 sugars, including Melezitose, Xylitol, Cellobiose, D-Arabinose, Sorbose, Rhamnose, alpha-Methyl-D-Mannoside, Malonate, Esculin, Citrate, O-Nitrophenyl-β-D-galactopyranoside (ONPG) and one control. Parts C of the wells in the kit were inoculated similarly to the *Actinomycetes* and incubated at 30 °C for 7–14 days [33].

##### Starch Utilization Test

A starch hydrolysis test was performed in which the isolates were inoculated on sterile starch agar plates and incubated at 30 °C for seven days. Following the incubation period, iodine solution was flooded into the plates. Starch hydrolysis was confirmed by the presence of a clear zone of hydrolysis around the bacterial growth [27].

##### Gelatin Hydrolysis Test

In the gelatin hydrolysis test, the isolates were inoculated on gelatin agar plates and incubated at 30 °C for seven days to check the gelatin hydrolysis. At the end of the incubation period, 1 mL mercuric chloride solution was dispersed into the agar medium to observe the zone of hydrolysis [27].

##### Casein Hydrolysis Test

The isolates were similarly streaked on skimmed milk agar medium plates. They were then incubated at 30 °C for seven days to see whether there was a zone of hydrolysis [27].

##### Citrate Utilization Test

The isolates were also inoculated on Simon’s citrate slant agar test tubes incubated at 30 °C for seven days until a color change was found [27].

#### 4.6.3. Physiological Characterization

To test the NaCl resistance, the AIA medium was prepared in seven batches and amended with 0%, 1%, 3.5%, 5%, 7%, 10% and 20% NaCl. The isolates were streaked on the media and incubated at 30 °C for seven days. The growth of the isolates at the highest salt concentration was observed and recorded.

The sterile AIA plates were inoculated with the isolates and incubated at 4 °C, 20 °C, 25 °C, 30 °C, 37 °C and 40 °C for seven days. The maximum growth at the optimal temperature was recorded by the visual observation of growth [50].

#### 4.6.4. Molecular Characterization

Based on the biochemical characteristics and morphology of the isolate, the isolate that showed good antibacterial activity was chosen for further study. The 16S ribosomal RNA (rRNA) sequence analysis was carried out to confirm the isolated bacterium.

### 4.7. Bacterial Cultures Used

The two bacterial fish pathogens selected for this study were *Vibrio parahemolyticus* (MTCC 451) and *Aeromonas hydrophila* (MTCC 1739). The stock and slant cultures of these test organisms were obtained from the Microbial Type Culture Collection (MTCC). Both cultures were inoculated into the nutrient broth and sub-cultured on nutrient agar plates before performing the test.

### 4.8. In Vitro Antibacterial Activity

#### 4.8.1. Primary Screening

Preliminary screening was carried out by the cross-streak method in which the isolates were screened against the selected test pathogens. Mueller–Hinton Agar (MHA) plates were prepared and supplemented with 2% sodium chloride. The *Actinomycete* isolates were inoculated with a single streak in the center of MHA plates and incubated at 30 °C for 7 days. Later, the sub-cultured test cultures were streaked perpendicular to the *Actinomycetes* isolates at a 90° angle and incubated at 37 °C for 24 h. The zone of inhibition was recorded [35]. Based on the zone of inhibition observed against the test organism, the potential isolates of *Actinomycetes* were selected for secondary screening.

#### 4.8.2. Secondary Screening

The *Actinomycete* isolates that showed prospective antibacterial activities in primary screening were subjected to solvent extraction to obtain the crude extracts. The isolates were inoculated into a Starch Casein broth/ISP2 broth and kept in a rotary shaker incubator for seven days at 30 °C and 120 rpm until growth was visible. Centrifugation was performed at 4 °C at 10,000 rpm for 20 min, and the filtrate was aseptically transferred into conical flasks. Equal volumes of organic solvents with ethanol and an aqueous extract with distilled water were added to the cell-free supernatant (CFS) and shaken well for two hours to extract the antibacterial compounds [51].

##### Agar Well Diffusion Method

The antibacterial activities of the crude extracts were tested at different concentrations, ranging from 50 µg/mL to 250 µg/mL, by the agar well diffusion method. Lawn cultures of the pathogenic organisms were spread on the solidified MHA agar plates using sterile cotton swabs. The wells were prepared on MHA plates using a sterile cork borer. The different concentrations of both ethanolic and aqueous extracts of *Actinomycete* isolates were poured into each well and allowed to diffuse completely. Culture plates were incubated at 37 °C for 24 h. Experiments were carried out three times, and the mean of the zone of inhibition (in mm) was recorded [52].

##### Disc Diffusion Method

Gentamycin (10 mcg) was taken for the antibiotic sensitivity test, which was performed for both pathogens. The *Actinomycete* isolate showing the maximum zone of inhibition was chosen for further study by the Kirby–Bauer agar disc diffusion method [36] to check the antibacterial activity against pathogens. In this method, the pathogens were first swabbed entirely from the surface of sterile MHA plates. Then, the concentration of the isolate showing positive results was added inside the disc and placed on the surface of the medium and incubated at 37 °C for 24 h. The zone of inhibition was recorded in triplicate to calculate the mean value of the inhibition zones.

### 4.9. Extraction of the Active Compound

The inoculum was prepared by inoculating the selected *Actinomycete* isolate into a Starch Casein Broth/ISP-2 Broth kept in a rotary shaker incubator at 30 °C and harvested after 7 days. The broth culture was then filtered through Whatman No. 1 filter paper. The filtrate obtained was centrifuged at 4 °C, 10,000 rpm, for 20 min. The CFS collected was further used for the extraction of metabolites. The CFS was extracted three times with an equal volume of ethyl acetate by shaking manually at a 1:1 ratio by using a separating funnel. Then, it was kept in a shaker for 48 h, after which it was removed and the solvent layer collected. The concentration of the solvent layer was carried out using a rotary evaporator to obtain the crude metabolites [33]. As per some earlier reports, studies were conducted using various solvents, which gave better results in the extraction of bioactive compounds from the CFS of *Actinomycetes*. Among them, ethyl acetate was found to be a good polar solvent [53,54].

### 4.10. Identification of the Crude Bioactive Compound

Compounds obtained from the *Actinomycete* isolate were dissolved in ethyl acetate and identified using gas chromatography–mass spectroscopy (GC-MS) and Fourier transform infrared spectroscopy (FTIR) analyses.

A 13 mm potassium bromide (KBr) powder was mixed and ground thoroughly using a mortar and pestle with the crude bioactive compounds isolated from potential *Actinomycete* isolates. The sample was placed inside a pellet dye in a quantity just sufficient to cover its bottom. It was pressed at 5000–10,000 psi using the pellet press to form a pellet disc and placed in the sample holder. It was then analyzed by FTIR to obtain the measurement in the spectrum wave number range between 4 and 4000/cm at a resolution of 4 cm^−1^ using a FTIR spectrometer (Jasco, Victoria, BC, Canada).

GC-MS was performed for the identification of the active components present in the ethanolic extract of *Actinomycetes.* The unknown components were identified using a GC-MS (Agilent 6890/Hewlett-Packard 5975 (Agilent, Palo Alto, CA, USA/Hewlett Packard Labs, Milpitas, CA, USA) under the electron impact (EI) mode. Several acquisition parameters were followed with respect to the analysis. The interpretation of the mass spectrum was carried out using the National Institute Standard and Technology (NIST) database. The NIST library was used for searching for the spectrum of unknown compounds from the *Actinomycetes* [55].

## 5. Conclusions

Sixteen *Actinomycetes* were successfully isolated from marine sediments. Among them, two strains displayed antibacterial activity, but one strain, *Beijerinickia fluminensis,* was reported to have potent antibacterial activity and was hence selected for further analysis. FTIR and GC-MS revealed several antibacterial compounds that can be used as an alternative to antibiotics for the treatment of several pathogens in aquaculture. Based on the literature survey, this might be the first report on the isolation of *Beijerinickia fluminensis* from marine sediments.

## Figures and Tables

**Table 1:** Antibacterial activity of *Actinomycete* isolates compared to Gentamicin.

S.No.	Isolates	Antibacterial Activity/Zone of Inhibition (in mm)		Zone of Inhibition in Gentamicin (in mm)	
		*Aeromonas hydrophila*	*Vibrio parahemolyticus*	*Aeromonas hydrophila*	*Vibrio parahemolyticus*
1	Isolate 1 *(Beijernickia fluminensis)*	11	13	12	11
2	Isolate 2	9	12	12	11

**Table 2:** Biochemical tests of *Beijerinickia fluminensis* VIT01 strain.

Biochemical Characteristics	*Beijerinickia fluminensis*
ONPG	Positive
Lysine Utilization	Positive
Ornithine Utilization	Positive
Urease	Positive
Phenylalanine Deamination	Negative
Nitrate Reduction	Positive
H_2_S Production	Negative
Citrate Utilization	Positive
Voges–Proskauer	Negative
Methyl Red	Negative
Indole	Negative
Malonate Utilization	Negative
Esculin Hydrolysis	Positive
L-Arabinose	Negative
Xylose	Negative
Adonitol	Negative
Rhamnose	Negative
Cellobiose	Negative
Melibiose	Negative
Saccharose	Positive
Raffinose	Negative
Trehalose	Positive
Glucose	Positive
Lactose	Positive
Oxidase	Negative
Casein Utilization	Positive
Melezitose	Negative
α–Methyl–D-Mannoside	Negative
Xylitol	Positive
D-Arabinose	Negative
Sorbose	Positive

**Table 3:** FTIR analysis of compounds exhibiting their functional groups.

Wavelength	Functional Group/Bonds	Band Strength	Compound
*Ethyl Acetate Extract of Actinomycetes (Sample)*			
2652.15	S-H stretch	Weak	Thiol
2573.22	O-H stretch	Strong, Broad	Carboxylic acid
2041.57	N=C=S stretch	Strong	Isothiocyanate
2013.71	N=C=S stretch	Strong	Isothiocyanate
1292.27	C-F stretch	Strong	Fluoro compound
1167.49	C-O stretch	Strong	Tertiary alcohol
1097.84	C-N stretch	Medium	Amine
1042.705	C-N stretch	Medium	Amine
991.63	C=C bending	Strong	Alkene
885.41	C=C bending	Strong	Alkene
751.34	C-H bending	Strong	1,2-disubstituted
709.55	C=C bending	Strong	Alkene
556.909	C-Cl stretching	Strong	Halo compound
464.62	C-l stretching	Strong	Halo compound

**Table 4:** Activity of bioactive compounds found in ethyl acetate extract of *Beijerinickia fluminensis*.

S.No	Compounds	Major Peak Area (%)	Retention Time (RT)	Major Activities * Present in Sample
1	N, N-Dimethylheptanamide	28	2.64	Antibacterial, Antitumor
2	Butanamide,3,N-Dihydroxy	273	3.86	Antitumor
3	3-O-Acetyl-Exo-1,2-O-Ethylidene- Alpha-D-Erythrof	785	6.42	Anticancer
4	5-Aminovaleric acid	1438	9.69	Anticarcinogenic
5	Glycine,N-Octyl-,Ethyl Ester	2437	14.69	Antibacterial, Antitumor
6	As-Triazine-3,5(2H,4H)-Dione, 6-(Dimethylamino)	2895	16.98	
7	Glycyl-L-Proline	3006	17.53	Antibacterial
8	Propyl Aldoxime, 2-Methyl-, Syn	3123	18.12	DNA synthesis inhibitor
9	Actinomycin C2	3368	19.35	Antibacterial, Anticancer
10	(S)-3,4-Dimethylpentanol	3411	19.56	Antibacterial, Anticancer, Antidiabetic, DNA synthesis inhibitor
11	7-Tetradecene, (E)	3767	21.34	Antibacterial, Anticancer
12	2-Decenioc acid	3844	21.73	Anticarcinogenic
13	1-Decene, 8-Methyl	4234	23.68	Methyl-guanidine inhibitor
14	Heneicosane, 11-Phenyl	4442	24.72	-
15	Dodecane, 1-ChloroHeneicosane, 11-Phenyl	4757	26.29	-
16	Dichloroacetic Acid, 2-Ethylhexyl Ester	4780	26.41	-
17	Pterin-6-Carboxylic acid	4942	27.22	Anticarcinogenic
18	2,3-Anhydro-D-Galactosan	5378	29.40	Anticancer

* Dr. Duke’s Phytochemical and Ethnobotanical Database [25].

## Data Availability

Not applicable.
